# Ten recommendations for using implementation frameworks in research and practice

**DOI:** 10.1186/s43058-020-00023-7

**Published:** 2020-04-30

**Authors:** Joanna C. Moullin, Kelsey S. Dickson, Nicole A. Stadnick, Bianca Albers, Per Nilsen, Sarabeth Broder-Fingert, Barbara Mukasa, Gregory A. Aarons

**Affiliations:** 1 Faculty of Health Sciences, School of Pharmacy and Biomedical Sciences, Curtin University, Kent Street, Bentley, Søborg, Western Australia 6102 Australia; 2 Child and Adolescent Services Research Center, 3665 Kearny Villa Rd., Suite 200N, San Diego, CA 92123 USA; 3 San Diego State University, 5500 Campanile Drive, San Diego, CA 92182 USA; 4 Department of Psychiatry, University of California San Diego, 9500 Gilman Drive (0812), La Jolla, CA 92093-0812 USA; 5 UC San Diego Dissemination and Implementation Science Center, 9452 Medical Center Dr, La Jolla, CA 92037 USA; 6 European Implementation Collaborative, Odense, Denmark; 7 School of Health Sciences, University of Melbourne, 161 Barry St, Carlton, VIC 3053 Australia; 8 Department of Health, Medicine and Caring Sciences, Linköping University, 58183 Linköping, Sweden; 9 School of Medicine, Department of Pediatrics, Boston Medical Center and Boston University, 801 Albany Street, Boston, MA 02114 USA; 10 Mildmay Uganda, 24985 Lweza, Entebbe Road, Kampala, Uganda

**Keywords:** Implementation, Frameworks, Models, Theory

## Abstract

**Background:**

Recent reviews of the use and application of implementation frameworks in implementation efforts highlight the limited use of frameworks, despite the value in doing so. As such, this article aims to provide recommendations to enhance the application of implementation frameworks, for implementation researchers, intermediaries, and practitioners.

**Discussion:**

Ideally, an implementation framework, or multiple frameworks should be used prior to and throughout an implementation effort. This includes both in implementation science research studies and in real-world implementation projects. To guide this application, outlined are ten recommendations for using implementation frameworks across the implementation process. The recommendations have been written in the rough chronological order of an implementation effort; however, we understand these may vary depending on the project or context: (1) select a suitable framework(s), (2) establish and maintain community stakeholder engagement and partnerships, (3) define issue and develop research or evaluation questions and hypotheses, (4) develop an implementation mechanistic process model or logic model, (5) select research and evaluation methods (6) determine implementation factors/determinants, (7) select and tailor, or develop, implementation strategy(s), (8) specify implementation outcomes and evaluate implementation, (9) use a framework(s) at micro level to conduct and tailor implementation, and (10) write the proposal and report. Ideally, a framework(s) would be applied to each of the recommendations. For this article, we begin by discussing each recommendation within the context of frameworks broadly, followed by specific examples using the Exploration, Preparation, Implementation, Sustainment (EPIS) framework.

**Summary:**

The use of conceptual and theoretical frameworks provides a foundation from which generalizable implementation knowledge can be advanced. On the contrary, superficial use of frameworks hinders being able to use, learn from, and work sequentially to progress the field. Following the provided ten recommendations, we hope to assist researchers, intermediaries, and practitioners to improve the use of implementation science frameworks.

Contributions to the literature

- Provision of recommendations and concrete approaches to enhance the use of implementation science frameworks, models, and theories by researchers, intermediaries, and practitioners
- Increase the ability of implementation researchers to produce generalizable implementation knowledge through comprehensive application of implementation frameworks, models, and theories
- Increase implementation intermediaries and practitioners ability to use implementation frameworks as a shared language to familiarize stakeholders with implementation and as practical tools for planning, executing, and evaluating real-world implementation efforts
- Provision of a worksheet to assist the application our recommendations for comprehensive framework use
- Provision of a checklist to assist in reviewing ways in which the selected framework(s) are used

## Background

There is great value in effectively using implementation frameworks, models, and theories [1, 2]. When used in research, they can guide the design and conduct of studies, inform the theoretical and empirical thinking of research teams, and aid interpretation of findings. For intermediaries and practitioners, they can provide shared language to familiarize stakeholders with implementation and function as practical tools for planning, executing, and evaluating real-world implementation efforts. Implementation frameworks, models, and theories have proliferated, and there are concerns that they are not used optimally to substantiate or advance implementation science and practice.

Theories are generally specific and predictive, with directional relationships between concepts making them suitable for hypothesis testing as they may guide what may or may not work [3]. Models are also specific in scope, however are more often prescriptive, for example, delineating a series of steps. Frameworks on the other hand tend to organize, explain, or describe information and the range and relationships between concepts, including some which delineate processes, and therefore are useful for communication. While we acknowledge the need for greater use of implementation frameworks, models, and potentially even more so theories, we use the term frameworks to encompass the broadest organizing structure.

Suboptimal use of frameworks can impact the viability and success of implementation efforts [4]. This can result in wasted resources, erroneous conclusions, specification errors in implementation methods and data analyses, and attenuated reviews of funding applications [5]. There can be a lack of theory or poorly articulated assumptions (i.e., program theory/logic model), guiding which constructs or processes are involved, operationalized, measured, and analyzed. While guidance for effective grant applications [4] and standards for evaluating implementation science proposals exist [6], the poor use of frameworks goes beyond proposals and projects and can slow or misguide the progress of implementation science as a field. Consistent terms and constructs aid communication and synthesis of findings and therefore are keys to replication and to building the evidence base. In real-world practice, the suboptimal use of implementation frameworks can lead stakeholders to misjudge their implementation context or develop inappropriate implementation strategies. Just as important, poor use of frameworks can slow the translation of research evidence into practice, and thereby limit public health impact.

Frameworks are graphical or narrative representations of the factors, concepts, or variables of a phenomenon [3]. In the case of implementation science, the phenomenon of interest is implementation. Implementation frameworks can provide a structure for the following: (1) describing and/or guiding the process of translating effective interventions and research evidence into practice (process frameworks), (2) analyzing what influences implementation outcomes (determinant frameworks), and (3) evaluating implementation efforts (outcome frameworks) [2]. Concepts within implementation frameworks may therefore include the following: the implementation process, often delineated into a series of phases; factors influencing the implementation process, frequently referred to as determinants or barriers and facilitators/enablers; implementation strategies to guide the implementation process; and implementation outcomes. The breadth and depth to which the concepts are described within frameworks vary [7].

Recent analyses of implementation science studies show suboptimal use of implementation frameworks [1, 8]. Suboptimal use of a framework is where it is applied conceptually, but not operationalized or incorporated throughout the phases of an implementation effort, such as limited use to guide research methods [1, 9]. While there is some published guidance on the use of specific frameworks such as the Theoretical Domains Framework (TDF) [10], RE-AIM [11], the Consolidated Framework for Implementation Research (CFIR) [12], the Exploration, Preparation, Implementation, Sustainment (EPIS) framework [1], and combined frameworks [13], there is a need for explicit guidance on the use of frameworks generally. As such, this article provides recommendations and concrete approaches to enhance the use of implementation science frameworks by researchers, intermediaries, and practitioners.

## Recommendations for using implementation framework(s)

Ideally, implementation frameworks are used prior to and throughout an implementation effort, which includes both implementation research and real-world implementation projects. Described below, we present ten recommendations for the use of implementation frameworks, presented in the rough chronological order of an implementation effort. The sequence is not prescriptive to accommodate flexibility in project design and objectives; the order of recommendations one to three in particular may vary or occur concurrently. The key is that all recommendations are considered and that ideally a framework(s) would be applied to each recommendation. This may mean one framework is used across all recommendations or multiple frameworks are employed. We recognize that this may be unrealistic when working under real-world resource constraints and instead strategic selection of frameworks may be necessary (e.g., based on the greatest needs or strongest preferences of stakeholders).

Depending on the stage in the implementation process, it may not be necessary to apply all the recommendations. The full list is suitable for implementation efforts that will progress at least to the implementation stage, whereby implementation strategies are being employed. However, for those who are early in the exploration phase of implementation or perhaps at the point of trying to establish implementation determinants, they may not be able to produce process or logic models or articulate mechanisms yet. This does not mean a framework is not very informative, but the order of the recommendations would vary and the full list may only be applicable as the implementation project progresses in future work.

We begin by discussing each recommendation within the context of frameworks broadly, followed by specific examples using the EPIS framework. The EPIS framework acknowledges the dynamic nature of implementation by defining important outer context, inner context, bridging, and innovation factors that influence or are influenced by an implementation effort throughout the phases of implementation. These applied examples are based on the results of a recent systematic review [1], and the collective experience of the co-authors applying the EPIS framework in national and international implementation efforts. In addition, we provide two tools that summarize each recommendation along with key questions to consider for optimal framework application within research, evaluation, and practice projects (Additional files 1 and 2).

To ensure that the recommendations are clear, practical, and comprehensive, we invited an international stakeholder panel who come from different perspectives (e.g., researcher, NGO administrator, intermediary, provider/physician) to review the recommendations and consider their utility applied to their implementation efforts. Our four-member panel included at least one stakeholder from each target audience for this article including implementation researchers, whose work spans diverse contexts, populations, and academic disciplines; evidence-based practice (EBP); intermediaries; and practitioners. Stakeholders reported extensive applied and training experience using multiple frameworks (e.g., CFIR and the Capability, Opportunity, Motivation (COM-B) component of the Behaviour Change Wheel (BCW)). Specifically, the goal of the stakeholder input was to critically review the paper, making any additions, edits, and comments, by concentrating their thinking on (i) Would they be able to apply these recommendations as they are written to their implementation work (proposals, studies, projects, evaluations, reports etc.)? (ii) Would they as a researcher, administrator, intermediary, or provider know what to do to use an implementation framework for each recommendation? In addition, we felt one area that needed some extra attention was the two tools, which aim to assist readers apply the recommendations. They were asked to test/trial the tools with any projects that they or a colleague had to ensure they were functional. The tools were refined according to their suggestions.

### Select a suitable framework(s)

The process for selecting implementation framework(s) for a particular implementation effort should consider the following: (i) the purpose of the framework (describing/guiding the implementation process, analyzing what influences outcomes [barriers and facilitators], or evaluating the implementation effort); (ii) the level(s) included within the framework (e.g., provider, organization, system); (iii) the degree of inclusion and depth of analysis or operationalization of implementation concepts (process, determinants [barriers and facilitators], strategies, evaluation); and (iv) the framework’s orientation, which includes the setting and type of intervention (i.e., EBP generally, a specific intervention, a guideline, a public health program being implemented) for which the framework was originally designed [7]. Reviews and websites of implementation frameworks provide lists of potential options [1, 2, 14, 15], and the Theory Comparison and Selection Tool (T-CaST) defines specific framework selection criteria [16]. Frameworks may be evaluated against these four criteria to see if they fit the implementation effort’s purpose (aims and objectives) and context (setting in which implementation is to occur). If for example a project was aiming to implement an educational program in a school setting, a framework that includes factors associated with the healthcare system or patient characteristics would not be a good fit.

It may be necessary and desirable to use multiple frameworks. Confusing matters, some frameworks fit neatly within one framework category, while others cross multiple framework “types.” For example, EPIS is both a process as well as a determinant framework with its focus on inner and outer context determinants across the phases of implementation. Furthermore, frameworks include different concepts and operationalize these to varying degrees. Put simply, some frameworks are more general, while others are more context or intervention specific; some frameworks are more comprehensive than others. Selecting a given framework can simultaneously expand and limit consideration of factors and processes likely to be important in an implementation effort. For expansion, frameworks can enumerate issues that might not have been considered for a given effort. On the other hand, limiting consideration of implementation issues to only the theories, constructs, and/or processes identified in a given framework may attenuate or curtail the degree to which factors affecting implementation are considered. Thus, it is sometimes desirable to use multiple frameworks for specific purposes, or alternatively expand on a current framework. For example, researchers may use a framework for understanding and testing determinants (e.g., EPIS [17], CFIR [18], TDF [10, 19, 20]) and another for evaluating outcomes (e.g., RE-AIM [21] or Proctor’s [22]).

Finally, we recommend that framework users invest in knowledge of the service setting in which they are working. This includes knowing or seeking involvement from stakeholders who understand the external context such as community norms and culture, policy and government processes, as well as the inner context such as organizational culture and climate, employee expectations, and attitudes towards innovations. Framework use in isolation without a deep understanding of context specific issues can result in a mismatch between framework selection and its applicability in research and practice. Furthermore, it is vital to seek permissions from both inner context and external context leadership.

#### EPIS application

A mixed-methods developmental project aimed to systematically adapt and test an EBP for youth with Autism Spectrum Disorder in publicly-funded mental health settings and develop a corresponding implementation plan [23]. EPIS was specifically selected by the research team, given the EPIS framework’s focus on public services settings, that it specifies multi-level inner and outer contextual factors, bridging factors between outer and inner contexts, addresses implementation process, and emphasizes innovation fit. EPIS was an apt fit for the project aims and context. In combination with the EPIS framework and as one example of a bridging factor, a community partnership model [24] was also applied to inform the community-academic partnership integrated throughout this study.

### Establish and maintain community stakeholder engagement and partnerships

Stakeholder engagement is an integral component of implementation [25, 26]. Growing calls are being made for [27] and examples of embedded research models, such as practice-based research networks, learning health systems, and implementation laboratories [28], that foster collaborations between researchers, implementers, and policy-makers integrated within a healthcare system to conduct research. Frameworks help inform discussions related to the types and specific roles of stakeholders who should be engaged, and the timing of stakeholder engagement. Stakeholders should not only include those who are proximally involved in EBP service delivery and receipt (consumers, providers, and administrative staff), but also those who are distally involved in oversight and structuring organizations, legislative actions, policy design, and financing of EBP delivery [29]. Engaging stakeholders across multiple levels of an implementation ecosystem (e.g., policy/legislative, funders, community, organizational, provider, client/patient) is recommended best practice for implementation researchers [30] and as indicated in the multi-level nature of the majority of implementation frameworks. Implementation frameworks generally encourage stakeholder engagement prior to funding, and for it to continue during implementation effort justification and as part of future implementation iterations and adaptations. Further, an implementation framework can inform clarity. Stakeholders can be engaged in the application of an implementation framework by, for example, having them involved in defining the local health system needs and selecting EBP(s) and/or implementation strategies in the EPIS implementation phase, as these are important to enhance their collaboration and ownership of the implementation effort [26].

Several implementation and improvement science frameworks explicitly include stakeholder engagement as a key construct or process (e.g., EPIS framework, PRECEDE-PROCEED, Plan-Do-Study-Act cycles, Promoting Action on Research Implementation in Health Services [PARIHS]). Additionally, there are pragmatic tools drawn from frameworks that can facilitate stakeholder engagement. For example, key criteria within the aforementioned T-CaST tool include the extent to which stakeholders are able to understand, apply, and operationalize a given implementation framework, and the degree to which the framework is familiar to stakeholders [16]. Methods, such as concept mapping [31], nominal group technique [32], and design thinking [33], may be used to guide stakeholder engagement meetings and define the issue or gap to be addressed. Other frameworks, such as the BCW [34], EPIS [17], or CFIR [18], may be used to prioritize and define implementation outcomes, determinants, and strategies together with stakeholders.

#### EPIS application

The EPIS framework explicitly highlights the importance of engaging multiple levels of stakeholders to influence implementation efforts longitudinally and contextually, from the initial identification of a need to sustainment of EBP delivery to address that need. While duration or depth of stakeholder engagement is not explicitly prescribed in EPIS, if combined with, for example, a designated partnership engagement model [24], EPIS has shown to enable the conceptualization and characterization of roles and levels of stakeholder engagement (system leaders program managers, providers) within system-driven implementation efforts [35].

### Define issue and develop research or evaluation questions and hypotheses

Use of frameworks to inform the articulation of an implementation need (i.e., a research-practice gap) and the development of practice-related or research questions and hypotheses has the potential to optimize implementation efforts and outcomes [2]. Specifically, frameworks facilitate the framing and formulation of implementation questions, including those related to needs assessment (e.g., what is the clinical or implementation issue needing to be addressed?), process (e.g., what phases will the implementation undergo to translate an intervention into practice, or when is an organization ready to implement a new intervention?), implementation effectiveness (e.g., do the proposed implementation strategies work in the local context?), mechanisms of success (e.g., did an increase in implementation climate improve implementation intentions?), and associated impact on outcomes (e.g., how did the implementation effort perform in terms of adoption or reach?). Ideally, these questions—be they related to research projects or practice issues that providers want to resolve—should be closely linked with the framework selected to maximize impact. For example, the selection of the BCW as a guiding framework necessitates for a question or issue to be described in behavioral terms and, in many cases, refined to be more specific. Being specific about the problem to be addressed entails being precise about the behaviors you are trying to change and whose behavior is involved [36].

Frameworks also provide guidance for the translation of implementation literature to research or evaluation questions. For example, it has been written that education used alone as a single implementation strategy is not sufficient for successful implementation. An implementation framework will assist in realizing implementation determinants that remain to be addressed and therefore the selection of additional implementation(s) strategies. This can be challenging given the presence of multiple factors spanning different levels that vary across contexts and phases of implementation. Further, they contextualize and provide critical links between theory and individual experience gained through practice, such as supporting the perceived value of targeting leadership in promoting the adoption and use of effective interventions or research evidence [37].

Finally, and perhaps most relevant to many implementation efforts, frameworks provide explicit guidance and justification for proposed hypotheses to be tested that strengthen proposals, projects, trials, and products, both research and practice based [2, 4]. Despite its explanatory power, use of frameworks to explicitly guide hypothesis formation are the minority, even within implementation efforts using theory to guide other aspects of the research process [38–40]. Thus, the increased use of frameworks to inform implementation questions and hypotheses is sorely needed.

#### EPIS Application

Work by Becan and colleagues [41] provides an example of a comprehensive application of EPIS framework to inform hypothesis development in their US National Institute on Drug Abuse study Translational Research on Interventions for Adolescents in the Legal System (JJ-TRIALS). JJ-TRIALS utilized EPIS to inform, identification of outer and inner context determinants, measures to assess those determinants, predictions based on theory, and tracking progress through the EPIS phases including identifying what constitutes the transition between each phase and the next phase. Specifically, the trial applied EPIS to inform the development of four tiers of questions related to the following: (1) the differential effect of two implementation strategies, (2) the factors that impacted and supported the transition across implementation phases, (3) the impact of this process on key implementation outcomes, and (4) tracking progress through the EPIS phases. For example, relevant determinants at the outer context system level and inner context organizational levels were identified. Specific hypotheses were developed to test how determinants (e.g., independent variables) influenced mechanisms (e.g., mediators/moderators) and ultimately “targets” (e.g., dependent variables) that are implementation outcomes and outcomes with clinical relevance.

### Develop implementation program theory or logic model

Within research and practice projects, implementation frameworks can inform the program logics that describe the anticipated relationships between inputs, activities, outputs, and implementation and client outcomes, thereby supporting the explicit formulation of key assumptions and outlining of crucial project details.

In addition, implementation frameworks guide the design of a model for testing, for example, mediation and moderation of various influences on the process and outcomes of implementation. Despite an increasing emphasis on understanding key mechanisms of change in implementation [4, 42, 43], few evaluations examine implementation change mechanisms and targets [44]. Change mechanisms explain how or why underlying processes create change, whereas targets are defined as the identified focus or end aim of implementation efforts [45]. From a public health perspective, mechanism and target evaluation is critical to facilitate replication and scaling up of implementation protocols to more effectively change healthcare practice and achieve broader public health impact. Mechanism measurement and evaluation is critical to increase the rigor and relevance of implementation science [46]. Frameworks can facilitate beyond simple evaluation of key determinants and highlight fundamental single-level (e.g., organizational characteristics, individual adopter characteristics) and cross-cutting mechanisms of change spanning context or setting, levels [4]. Frameworks also enlighten the complex and evolving nature of determinants, mechanisms, and targets, varying across implementation phases. As an example, leadership may determine organizational climate during implementation within one specific service setting or context but serve as change mechanism impacting implementation targets during the exploration phase in a different setting. Frameworks provide the necessary roadmap for understanding these complex associations by offering prescriptive guidance for the evolving nature of these determinants.

#### EPIS Application

**Fig. 1:**
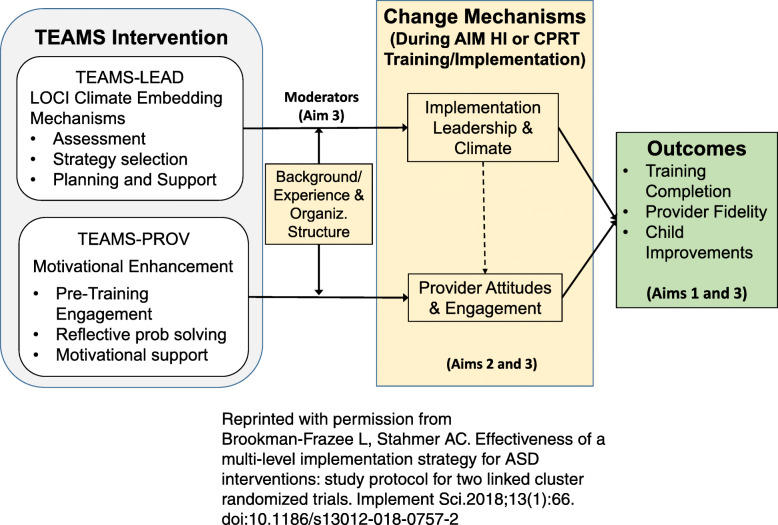
TEAMS intervention, mechanisms, and outcomes [47]

The EPIS framework was applied to predict implementation leadership and climate and provider attitudes as key mechanisms of change in two linked Hybrid Type 3 cluster randomized trials testing the effectiveness of multi-level implementation strategies targeting leadership and attitudes (Brookman-Frazee and Stahmer [47]; see Fig. 1). Consistent with the explanatory nature of EPIS, this work highlights the interconnected nature of these mechanisms, with leadership hypothesized as both a mechanism impacting outcomes as well as the predictor (determinant) of further mechanisms such as provider attitudes during implementation [47].

### Determine research and evaluation methods (overall design, data collection, data analysis)

The distinct aims and purposes of implementation efforts require distinct evaluation designs such as mixed-methods, hybrid effectiveness-implementation, and quality improvement approaches including formative evaluations or Plan-Do-Study-Act cycles [48]. Implementation frameworks should be used to inform development of such designs across all phases, from the broader construction down to the measurement and analysis.

In the design of an evaluation, frameworks should be used to inform decisions about what constructs to assess, data to collect, and which measures to use. In this process, frameworks can help to identify and/or expand the implementation determinants or aspects assumed to impact the implementation process at different levels and across multiple phases for consideration or measurement. They can also help to operationalize constructs of importance to an evaluation and the identification of suitable measures. Fortunately, there is expanding work in implementation science to develop and catalog tools tied to existing frameworks to aid in this application (e.g., EPIS, see episframework.com/measures [1]; CFIR, see cfirguide.org/evaluation-design [49]; RE-AIM, see re-aim.org/resources-and-tools [50]).

For the collection and analysis of qualitative data, frameworks such as EPIS or CFIR provide developed and freely available data analytic tools, including pre-populated coding templates and data aggregation matrices [1, 49]. Again, the use of framework-informed tools permits better alignment of concepts examined with broader implementation science literature. Analytically, frameworks can inform decisions about sequencing and directionality of implementation processes and strategies. Beyond identifying and analyzing key implementation determinants, theory should be applied along with frameworks in order to describe important implementation determinants (e.g., independent variables), implementation mechanisms (e.g., mediators), and their associated impacts on implementation targets (e.g., dependent variables) across the phases of implementation processes.

#### EPIS Application

The EPIS framework was used to inform the development of key informant interviews and focus groups, and data coding and analytic procedures to capture the key outer and inner context and innovation factor influences across implementation phases of two large-scale community effectiveness trials [51]. Within the trials themselves, EPIS informed the selection of quantitative measures of inner context organizational and provider measures [52]. Such integrated and thorough framework use is needed to further build an integrated body of knowledge about effective implementation strategies.

### Determine implementation determinants

Implementation frameworks often include several implementation determinants (i.e., barriers and enablers) that have been found to influence implementation outcomes [1, 2]. Such lists of potential determinants are useful for exploratory work, for example, identifying key factors for applying an intervention in a particular context. This may occur early in an implementation process to guide implementation strategy selection or EBP adaptation, or further along to aid in the development of an implementation plan or in tailoring implementation strategies to support the EBP implementation or adaptation. The implementation science literature includes numerous examples of using frameworks in this manner across health contexts (see Birken et al. (2017) [13]; Helfrich et al. (2010) [53]). Examples of relevant determinant frameworks include the EPIS [1, 17], CFIR [18], integrated checklist to identify determinants of practice (TICD checklist) [54], TDF [19], and BCW [36].

Another important reason for assessing implementation determinants using a theoretical framework is to specify the target of the implementation effort. It is not possible or necessary for all determinants to be targeted. Often, due to funding or other constraints, it is important to consider individual beneficiaries and community or government needs in prioritizing which determinants to targets. For example, the BCW methodology guides users to conduct a thorough behavioral diagnosis using the COM-B and to then prioritize which behaviors to address. In research, changes to pre-specified determinants included in the protocol require amendments to be documented, justified, and possibly approved by a research ethics committee. Prospective framework application may also reveal different determinants and aid selection of particular influencing factors to target during subsequent implementation studies.

#### EPIS Application

The Leadership and Organizational Change for Implementation (LOCI) intervention employed the EPIS framework to select key implementation determinants to test in a large cluster RCT [55]. In this study, implementation leadership from first-level team leaders/managers, organizational climate and culture, implementation climate, and psychological safety climate were selected as determinants to test their influence on the fidelity of the EBP being implemented. In addition, to the developed implementation model and implementation strategy, EPIS was used to code qualitative data and select quantitative survey measures.

### Select and tailor, or develop, an implementation strategy(s)

Implementation frameworks are necessary for selecting, tailoring, or developing implementation strategies. Defined as methods or techniques to aid the adoption, implementation, sustainment, and scale-up of evidence-based public health or clinical interventions [8], implementation strategies are the linchpin of successful implementation efforts. Implementation strategies vary in purpose and complexity, ranging from discrete strategies [56] such as audit and feedback [57] to multifaceted, and often branded, strategies that integrate at least two discrete strategies, such as the Leadership and Organizational Change for Implementation (LOCI) intervention [37], Availability, Responsiveness and Continuity model (ARC) [58], Replicating Effective Programs (REP) [59], Getting to Outcomes (GTO) [60], and Quality Implementation Framework (QIF) [61]. Powell and colleagues have outlined four primary methods for matching implementation strategies to barriers (conjoint analysis, intervention mapping, concept mapping, group model building) [62]. Each approach is highly participatory but varies in strengths and weaknesses of application. Additionally, comprehensive framework(s) application can help address identified priorities (e.g., methods for tailoring strategies, specifying, and testing mechanisms) for enhancing the impact of implementation strategies [63]. Taxonomies of strategies, such as the Expert Recommendations for Implementing Change (ERIC) discrete strategies list [64], BCT [65], and EPOC checklist [66], are useful to promote uniform communication and synthesis across implementation science.

Following the identification and prioritization of important barriers and facilitators (see recommendation 5), an implementation framework can support the process of matching determinants to implementation strategies. For example, the PARIHS framework [67] can be used to identify critical evidentiary (e.g., patient experience, information from the local setting) and contextual (e.g., leadership, receptive context) elements that may impact EBP implementation. This evidentiary and contextual analysis is then used to develop or tailor implementation strategies, primarily focused on facilitation as the anchoring approach. Use of frameworks like PARIHS to guide selection and tailoring of implementation strategies may be particularly suitable for implementation efforts and settings that have a strong need for facilitation to support the engagement and participation of a wide range or number of stakeholders.

#### EPIS application

The EPIS framework and the Dynamic Adaptation Process (DAP) were used in a cluster randomized trial to implement school nursing EBPs in US high schools to reduce LGBTQ adolescent suicide [68]. The DAP [69] is a multicomponent implementation strategy directly drawn from the EPIS framework. The DAP uses an iterative, data-informed approach to facilitate implementation across each phase of EPIS. A critical and core component of the DAP is the creation of an Implementation Resource Team that is a multiple stakeholder collaborative designed to support implementation, data interpretation, and explicitly address adaptations during the implementation process. Within this study, the EPIS framework and the DAP were used to (1) inform the constructs measured in the multi-level needs assessment during the exploration phase, (2) support the identification of the stakeholders and activities involved in the Implementation Resource Team that was developed in the preparation phase, (3) guide the tracking and integration of adaptations to the EBP strategy training and delivery during the implementation phase, and (4) inform the constructs and measurement of the implementation outcomes in the sustainment phase.

### Specify implementation outcomes and evaluate Implementation

Implementation evaluation may include evaluation of progression through implementation stages, formative and summative evaluation of factors and strategies, as well as evaluation of the degree of implementation success as reflected in implementation outcomes. These may be measured at micro (individual), meso (team or organization), and macro (system) levels. Regardless of the particular scope and design of implementation evaluations, they should be informed by implementation frameworks.

As outlined by Nilsen et al. [2], there are a few implementation frameworks that have the expressed purpose of evaluating implementation, including RE-AIM [21], PRECEDE-PROCEED [70], and frameworks by Stetler et al. [71], Moullin et al. [72], and Proctor et al. [22]. Furthermore, there are particular implementation process measures such as the Stages of Implementation Completion (SIC), which may be used as both a formative and summative tool to measure the rate and depth of implementation [73]. Furthermore, there is an increasing number of measures of implementation determinants [74, 75] (e.g., implementation leadership [76], implementation climate [77, 78], or implementation intentions [79]). Evaluation of changes in these factors over time may be indicators of implementation success. While there are aforementioned specific evaluation frameworks, other frameworks also include evaluation elements to varying degrees [7]. For example, the conceptual framework for sustainability of public health programs by Scheirer and Dearing [80], the framework of dissemination in health services intervention research by Mendel et al. [81], and the integrated 2-phase Texas Christian University (TCU) approach to strategic system change by Lehman [82] include comprehensive evaluation of the influencing factors depicted in the corresponding frameworks. Frameworks that do not explicitly include measurement components can draw upon evaluation frameworks to work alongside and to determine which measures to select for each of the influencing factors chosen to be studied and the nominated implementation outcomes.

#### EPIS application

While the EPIS framework is not primarily an evaluation framework, its website includes a list of measures for quantitative analysis and definitions for qualitative work. After selecting implementation determinants and developing specific implementation questions and/or hypotheses, implementation measures should be selected for the chosen determinants as mediators of implementation success. In addition, measures of movement through the EPIS stages and measures of implementation outcomes may be included (e.g., fidelity). Both JJ-trials (Juvenile Justice—Translational Research on Interventions for Adolescents in the Legal System) [83] and the LOCI study [37] provide examples for using EPIS in implementation evaluation. From a practice perspective, teams should measure the baselines and periodically throughout the project to determine how the process measures and outcomes have improved over time. These evaluations help determine the rate of progress, which can inform improvements in other recommendations, such as recommendations 5 and 7.

### Use a framework(s) at micro level to conduct and tailor implementation

Implementation is a dynamic, context-specific process. Each layer of a context (e.g., organization, profession, team, individual) requires ongoing individual tailoring of implementation strategies. Implementation frameworks, therefore, should be used to guide the overarching implementation plan, and—at the micro level—processes such as site-specific implementation team creation, barrier and facilitator assessment, implementation planning, and goal setting. This may be done by formatively evaluating implementation determinants either qualitatively or quantitatively as described above and then using the results to select or adapt implementation strategies for the particular context. Stetler et al. [71] provide four progressive yet integrated stages of formative evaluation. Another method would be to conduct implementation barrier, and facilitator assessments at different levels within the implementation context and subsequently determine tailor the implementation strategies. For example, coaching calls may reveal that a range of different behavioral change techniques [34] suited to each provider or leader.

#### EPIS application

During the aforementioned LOCI study, the goal was to improve first-level leader’s leadership and implementation climate to facilitate EBP adoption and use [55]. Baseline and ongoing 360-degree evaluation (where individuals, such as mid-level managers, rate themselves and receive ratings from their boss and staff) were performed and implementation plans subsequently adapted for each agency and team leader based on the data and emergent issues in the implementation process. This process was broadly informed by the focus on innovation fit and emphasis on leadership across levels within the EPIS framework. The Climate Embedding Mechanisms [84] were then used in combination with EPIS to formulate the individual, leader-specific implementation plans.

### Write the proposal and report

Documenting an implementation effort—be it in the form of a research proposal, a scientific article, or a practice report—is key for any project. As part of this documentation, detailing the use of an implementation framework(s) is vital for the implementation project to be replicable and analyzable. The use of the selected implementation framework(s) should be documented across the proposal and report. This includes description or selection of appropriate methods to assess the selected implementation determinants. Furthermore, as outlined by Proctor et al. [8], implementation strategies should be named, defined, and specified, based on seven components enabling their measurement and replication: actor, action, action targets, temporality (when), dose (duration and how often), outcomes, and theory/justification. Similarly, outcomes should be named, specified, measured, and reported. Again, the work of Proctor and colleagues [22] provides a useful taxonomy for classifying and reporting types of implementation research outcomes that also includes guidance regarding level of analysis and measurement, theoretical basis, and maps the salience of outcome onto the phases of implementation.

Consistent with these recommendations are existing standards and guidelines to improve transparent and accurate reporting of implementation studies such as the Standards for Reporting Implementation Studies (STaRI; Pinnock et al. [85]). Ideally, incorporating these standards will strengthen the comprehensive use and reporting of frameworks to inform the formulation, planning, and reporting of implementation studies. Our recommendation is to explicitly document the use of implementation frameworks in research proposals, scientific outputs, and evaluation reports. To aid this process, Additional file 1 provides the Implementation Framework Application Worksheet to provide examples of key questions to assist implementation scientists and practitioners in applying our recommendations for comprehensive framework application. Finally, Additional file 2 provides the Implementation Framework Utilization Checklist to assist in thinking through and reviewing ways in which the selected framework(s) are used. In combination with the Implementation Framework Application Worksheet, the Checklist may inform revisions to a project (proposal, active project, or dissemination materials) and facilitate comprehensive framework application. Additionally, this Checklist may serve to provide documentation of implementation utilization (e.g., for inclusion in project proposals, reports, manuscripts).

#### EPIS application

An example of EPIS framework reporting is the “ATTAIN” (Access to Tailored Autism Integrated Care) study protocol [86]. Within this example, the authors display an adapted EPIS framework to highlight the unique outer (e.g., American Academy of Pediatrics recommendation for mental health screening) and inner context (e.g., organizational and technological capacity for innovation) determinants relevant to the phases of implementation included in the study (Exploration through Implementation). In addition, the authors describe how the unique contextual determinants and proposed implementation strategies (e.g., inter-organizational relationships among stakeholders) were conceptualized and to be measured across the study’s lifespan.

## Conclusion

The use of implementation frameworks provides a structure for describing, guiding, analyzing, and evaluating implementation efforts, thus facilitating advancement of generalizable implementation science knowledge. Superficial use of frameworks hinders researchers’ and practitioners’ learning and efforts to sequentially progress the field. By following the provided ten recommendations, we hope researchers, intermediaries, and practitioners will bolster the use of implementation science frameworks.

## Supplementary information

**Additional file 1:Table S1.** Implementation Framework Application Worksheet.

**Additional file 2:Table S2.** Implementation Framework Utilization Tool.

## Data Availability

Not Applicable

## Abbreviations

ARC: Availability, Responsiveness and Continuity model
ATTAIN: Access to Tailored Autism Integrated Care
BCW: Behaviour Change Wheel
CFIR: Consolidated Framework for Implementation Research
COM-B: Capability, Opportunity, Motivation - Behaviour
DAP: Dynamic Adaptation Process
EBP: Evidence-Based Practice
EPIS: Exploration, Preparation, Implementation, Sustainment framework
EPOC: Expert Recommendations for Implementing Change
GTO: Getting to Outcomes
JJ-Trials: Juvenile Justice—Translational Research on Interventions for Adolescents in the Legal System
LOCI: Leadership and Organizational Change Intervention
PARIHS: Promoting Action on Research Implementation in Health Services
QIF: Quality Implementation Framework
RE-AIM: Reach, Effectiveness, Adoption, Implementation, Maintenance
REP: Replicating Effective Programs
STaRI: Standards for Reporting Implementation Studies
TCU: Texas Christian University
TDF: Theoretical Domains Framework
