# Supplementary material for: Ten recommendations for using implementation frameworks in research and practice
Source: Implement Sci Commun. 2020 Apr 30;1:42. doi: 10.1186/s43058-020-00023-7 (PMC7427911; doi:10.1186/s43058-020-00023-7)
Supplement: Supplementary file 1 — Additional file 1:Table S1. Implementation Framework Application Worksheet. [file 43058_2020_23_MOESM1_ESM.docx]

**Table S1. Implementation Framework Application Worksheet**

**Project title:** _______________________________________________________________________________

**Instructions:** For each recommendation, use this table to consider relevant questions to facilitate comprehensive application of the selected framework(s) across your implementation project. This is best done in collaboration with relevant stakeholders at the outset of a project and reviewed periodically. The sequence of the 10 recommendations is not designed to be strictly linear, as they will differ from project to project, and steps may also occur concurrently. Furthermore, depending on the project not all recommendations or questions may be relevant, in which case select not applicable (NA).

|  | **Recommendation** | **Framework(s) selected** | **Key Questions to Guide Application of Implementation Framework Recommendation** | |
| --- | --- | --- | --- | --- |
| 1 | Select appropriate implementation framework(s) |  | What is the purpose of the framework (e.g., guiding the process of implementation, analyzing the influences of implementation, or evaluating the implementation effort) and does this align with what is required for this implementation project? | Notes:  🞏 Not applicable |
|  |  |  | What level(s) does the framework address (provider, team, organisation, system) and does this align with the levels that are most relevant to this implementation project? | Notes:  🞏 Not applicable |
|  |  |  | What implementation concepts are included within the framework (process, determinants (barriers and facilitators), strategies, evaluation) and to what degree are they operationalized? | Notes:  🞏 Not applicable |
|  |  |  | How generalizable or specific is the implementation framework’s orientation (e.g., context, innovation, process)? | Notes:  🞏 Not applicable |
|  |  |  | Other questions | Notes: |

| 2 | Establish and maintain community stakeholder engagement and partnerships |  | How can the selected implementation framework(s) be used to inform which stakeholders are critical to (or not to) involve in this implementation project? | Notes:  🞏 Not applicable |
| --- | --- | --- | --- | --- |
|  |  |  | How can the selected implementation framework(s) suggest how to engage and involve stakeholders? | Notes:  🞏 Not applicable |
|  |  |  | How can the selected implementation framework(s) be used as a tool in engaging stakeholders in this implementation project? (i.e., as a simplified and therefore appealing and intuitive representation of the implementation process) | Notes:  🞏 Not applicable |
|  |  |  | How can the selected implementation framework(s) be used to determine the roles and activities of stakeholders within and across the implementation phase(s)? | Notes:  🞏 Not applicable |
|  |  |  | Other questions | Notes: |
| 3 | Define issue and develop research or evaluation questions and hypotheses |  | Which constructs from the selected framework can inform the research or evaluation questions and hypotheses? | Notes:  🞏 Not applicable |
|  |  |  | Can the questions or hypotheses help to advance the implementation framework(s) and/or implementation science knowledge? | Notes:  🞏 Not applicable |
|  |  |  | Other questions | Notes: |

| 4 | Develop implementation mechanistic process model or logic model |  | How can the selected framework(s) inform the design of a logic model for this implementation project? | Notes:  🞏 Not applicable |
| --- | --- | --- | --- | --- |
|  |  |  | How can the selected framework(s) inform the development of mechanisms of change (i.e., how can the selected framework(s) help to identify and describe the factors assumed to trigger the desired change of implementation behaviour in providers, teams, organisations, or systems)? | Notes:  🞏 Not applicable |
|  |  |  | Other questions | Notes: |
| 5 | Select research and evaluation methods |  | How can the selected implementation framework(s) inform the:   - Study / evaluation design? | Notes:  🞏 Not applicable |
|  |  |  | How can the selected implementation framework(s) inform the:   - Data collection tools? | Notes:  🞏 Not applicable |
|  |  |  | How can the selected implementation framework(s) inform the:   - Data analysis approaches? | Notes:  🞏 Not applicable |
|  |  |  | Other questions | Notes: |
| 6 | Determine implementation determinants (barriers and facilitators) |  | Which constructs included in the selected framework(s) represent determinants (barriers and facilitators) that we wish to study or that we anticipate will be of greatest importance to this implementation project? | Notes:  🞏 Not applicable |
|  |  |  | Other questions | Notes: |

| 7 | Select and tailor, or develop, an implementation strategy(s) |  | Given my selected implementation framework(s), the barriers and facilitators and mechanisms of change prioritised previously, which strategies are the most appropriate for this implementation project? | Notes:  🞏 Not applicable |
| --- | --- | --- | --- | --- |
|  |  |  | How are the selection and tailoring of implementation strategies consistent (or inconsistent) with the framework(s) selected? | Notes:  🞏 Not applicable |
|  |  |  | Other questions | Notes: |
| 8 | Specify implementation outcomes and evaluate implementation |  | Given the framework(s) selected, which implementation and other outcomes are key to measure as part of this implementation project? | Notes:  🞏 Not applicable |
|  |  |  | Given the framework(s) and determinants selected, what evaluation method and measures are being employed to measure determinants and changes in determinants over time? | Notes:  🞏 Not applicable |
|  |  |  | Given the framework(s) and strategies selected, what evaluation method and measures are being employed to assess the effectiveness of the implementation strategy? | Notes:  🞏 Not applicable |
|  |  |  | Given the framework(s) selected, how will we evaluate whether and how the implementation process progresses across different phases of the implementation being assessed? | Notes:  🞏 Not applicable |
|  |  |  | Given the framework(s) selected and the implementation and other outcomes selected, how should these be measured as part of the implementation evaluation? | Notes:  🞏 Not applicable |
|  |  |  | Other questions | Notes: |

| 9 | Use a framework(s) at micro level to conduct and tailor implementation |  | How can the selected implementation framework(s) inform tailoring and adaptation of the intervention to be implemented and the implementation strategies at all levels of the context (individuals, organization, system)? | Notes:  🞏 Not applicable |
| --- | --- | --- | --- | --- |
|  |  |  | Other questions | Notes: |
| 10 | Write the proposal and report |  | How can the selected implementation framework(s) guide and inform the project proposal and report(s)? | Notes:  🞏 Not applicable |
|  |  |  | How can the selected implementation framework(s) be incorporated throughout the project proposal and reports? (i.e., background, methods and results) | Notes:  🞏 Not applicable |
|  |  |  | Other questions | Notes: |
