# Supplementary material for: Ten recommendations for using implementation frameworks in research and practice
Source: Implement Sci Commun. 2020 Apr 30;1:42. doi: 10.1186/s43058-020-00023-7 (PMC7427911; doi:10.1186/s43058-020-00023-7)
Supplement: Supplementary file 2 — Additional file 2:Table S2. Implementation Framework Utilization Tool. [file 43058_2020_23_MOESM2_ESM.docx]

**Table S2: Implementation Framework Utilization Tool**

**Project Title:** ___________________________________________________________________________________

**Framework(s) considered/selected:** _________________________________________________________________

**Instructions:**  This tool is intended to assist in thinking through and reviewing ways in which the selected framework(s) are used. For each recommendation, note the extent to which each recommendation is applied. In combination with the Implementation Framework Application Worksheet, the tool may inform revisions to your project (proposal, active project or dissemination materials) to facilitate comprehensive framework application. Additionally, this tool may serve to provide documentation of implementation utilization (e.g., for inclusion in project proposals, reports, manuscripts). Depending on the project not all recommendations may be relevant, in which case select not applicable.

|  | **Recommendation** | **Framework(s)** | **Framework utilization** | |
| --- | --- | --- | --- | --- |
| 1 | Select appropriate framework(s) |  | The selected framework(s) cover all phases and aspects of the implementation project | 🞏 🞏 🞏 🞏 🞏  Not at Slight Moderate Great Not   all Extent Extent Extent Applicable |
|  | Establish and maintain community stakeholder engagement and partnerships |  | The selected implementation framework(s) are used to:  Select and define the roles of key stakeholders  Engage stakeholders across all phases of the implementation process | 🞏 🞏 🞏 🞏 🞏  Not at Slight Moderate Great Not  all Extent Extent Extent Applicable  🞏 🞏 🞏 🞏 🞏  Not at Slight Moderate Great Not  all Extent Extent Extent Applicable |
| 3 | Define issue and develop research or evaluation questions and hypotheses |  | Research and evaluation questions or hypotheses are derived from or consistent  with the selected implementation framework(s) | 🞏 🞏 🞏 🞏 🞏  Not at Slight Moderate Great Not  all Extent Extent Extent Applicable |
| 4 | Develop implementation mechanistic process model or logic model |  | The selected implementation framework(s) are the basis for:  The identification of mechanisms  A logic model | 🞏 🞏 🞏 🞏 🞏  Not at Slight Moderate Great Not  all Extent Extent Extent Applicable  🞏 🞏 🞏 🞏 🞏  Not at Slight Moderate Great Not  all Extent Extent Extent Applicable |

|  | **Recommendation** | **Framework(s)** | **Framework utilization** |  |
| --- | --- | --- | --- | --- |
| 5 | Select research and evaluation methods |  | The selected implementation framework(s) are used to inform:  Study /evaluation design  Data collection tools  Data analysis | 🞏 🞏 🞏 🞏 🞏  Not at Slight Moderate Great Not  all Extent Extent Extent Applicable  🞏 🞏 🞏 🞏 🞏  Not at Slight Moderate Great Not  all Extent Extent Extent Applicable  🞏 🞏 🞏 🞏 🞏  Not at Slight Moderate Great Not  all Extent Extent Extent Applicable |
| 6 | Determine implementation determinants |  | The key determinants selected for measurement and analysis are informed by  the selected implementation framework(s) | 🞏 🞏 🞏 🞏 🞏  Not at Slight Moderate Great Not  all Extent Extent Extent Applicable |
| 7 | Select and tailor, or develop, an implementation strategy(s) |  | The selection and tailoring of implementation strategies is consistent with the implementation framework(s) selected | 🞏 🞏 🞏 🞏 🞏  Not at Slight Moderate Great Not  all Extent Extent Extent Applicable |
| 8 | Specify implementation outcomes and evaluate implementation |  | The determinants chosen from the selected implementation framework(s)  are evaluated  The evaluation of the implementation strategy is informed by the selected implementation framework(s)  The movement across the implementation process/phases is informed by the selected implementation framework(s)?  The implementation outcomes measured are informed by the selected implementation framework(s) | 🞏 🞏 🞏 🞏 🞏  Not at Slight Moderate Great Not  all Extent Extent Extent Applicable  🞏 🞏 🞏 🞏 🞏  Not at Slight Moderate Great Not  all Extent Extent Extent Applicable  🞏 🞏 🞏 🞏 🞏  Not at Slight Moderate Great Not  all Extent Extent Extent Applicable  🞏 🞏 🞏 🞏 🞏  Not at Slight Moderate Great Not  all Extent Extent Extent Applicable |
| 9 | Use a framework(s) at micro level to conduct and tailor implementation |  | Implementation conduct and tailoring (e.g., selection of goals, team selection)  is informed by the selected implementation framework(s) | 🞏 🞏 🞏 🞏 🞏  Not at Slight Moderate Great Not  all Extent Extent Extent Applicable |
| 10 | Write the proposal and report |  | Project proposal and dissemination materials are guided by the  selected implementation framework(s) e.g., influence on methods and  evaluation are reported | 🞏 🞏 🞏 🞏 🞏  Not at Slight Moderate Great Not  all Extent Extent Extent Applicable |
